# Characterization of a potent non-cytotoxic shRNA directed to the HIV-1 co-receptor CCR5

**DOI:** 10.1186/1479-0556-7-8

**Published:** 2009-06-10

**Authors:** Saki Shimizu, Masakazu Kamata, Panyamol Kittipongdaja, Kevin N Chen, Sanggu Kim, Shen Pang, Joshua Boyer, F Xiao-Feng Qin, Dong Sung An, Irvin SY Chen

**Affiliations:** 1 Department of Hematology-Oncology, David Geffen School of Medicine and UCLA AIDS Institute, University California, Los Angeles, Los Angeles, California 90095, USA; 2 Department of Microbiology, Immunology and Molecular Genetics, David Geffen School of Medicine and UCLA AIDS Institute, University California, Los Angeles, Los Angeles, California 90095, USA; 3 University of California Los Angeles Dental Research Institute and University of California Los Angeles School of Dentistry, Los Angeles, California 90095, USA; 4 Department of Immunology, University of Texas MD Anderson Cancer Center, Houston, Texas 77030, USA

## Abstract

**Background:**

The use of shRNAs to downregulate the expression of specific genes is now relatively routine in experimentation but still hypothetical for clinical application. A potential therapeutic approach for HIV-1 disease is shRNA mediated downregulation of the HIV-1 co-receptor, CCR5. It is increasingly recognized that siRNAs and shRNAs can have unintended consequences such as cytotoxicities in cells, particularly when used for long term therapeutic purposes. For the clinical use of shRNAs, it is crucial to identify a shRNA that can potently inhibit CCR5 expression without inducing unintended cytotoxicities.

**Results:**

Previous shRNAs to CCR5 identified using conventional commercial algorithms showed cytotoxicity when expressed using the highly active U6 pol III promoter in primary human peripheral blood derived mononuclear cells. Expression using the lower activity H1 promoter significantly reduced toxicity, but all shRNAs also reduced RNAi activity. In an effort to identify shRNAs that were both potent and non-cytotoxic, we created a shRNA library representing all potential CCR5 20 to 22-nucleotide shRNA sequences expressed using an H1 promoter and screened this library for downregulation of CCR5. We identified one potent CCR5 shRNA that was also non-cytotoxic when expressed at a low level with the H1 promoter. We characterized this shRNA in regards to its function and structure. This shRNA was unique that the use of commercial and published algorithms to predict effective siRNA sequences did not result in identification of the same shRNA. We found that this shRNA could induce sequence specific reduction of CCR5 at post transcriptional level, consistent with the RNA interference mechanism. Importantly, this shRNA showed no obvious cytotoxicity and was effective at downregulating CCR5 in primary human peripheral blood derived mononuclear cells.

**Conclusion:**

We report on the characterization of a rare shRNA with atypical structural features having potent RNAi activity specific to CCR5. These results have implications for the application of RNAi technology for therapeutic purposes.

## Background

A finding with critical bearing upon HIV-1 disease was the fact that individuals homozygous for a defective CCR5 gene, CCR5Δ32, are protected from HIV infection and heterozygous individuals have a substantially prolonged course of disease[1,2]. If one could mimic the natural situation by genetic knockdown of CCR5, a potential therapy could be developed. The ultimate application of gene therapy for HIV-1 disease would be to introduce gene therapeutic elements as transgenes into a hematopoietic stem cell. Transplantation of such a stem cell would result in reconstitution of a hematopoietic system that in theory would be protected from the effects of HIV-1. The first step is the identification of effective reagents that can reduce CCR5 without unintended cytotoxicity.

Silencing of genes through homologous double stranded RNA is a sequence specific, highly conserved mechanism. It serves as an antiviral defense mechanism[3] and protects cells from retrotransposition[4,5]. siRNAs have been utilized experimentally to knock out gene expression from cellular and viral genes [6-10]. A RNA induced silencing complex (RISC) uses a siRNA as a guide sequence to cleave the target mRNA at the homologous sequence resulting in a decrease in the steady-state levels of target mRNA. Chemically synthesized siRNAs have been utilized to inhibit various virus infections including HIV-1[8,11]. siRNAs have also been expressed using plasmid vectors[6,9,12-14]. The antiviral effects of siRNA are sequence specific and differ from previously reported antisense mechanisms or to interferon and interferon response effectors protein kinase R (PKR) and RNaseL[15]. siRNA provides an attractive alternative to other gene therapeutic reagents due to its small size, and ease of manipulation. Although, the requirement for an effective siRNA are not completely understood, our experience and that of others indicate that choice of siRNAs based upon published guidelines[6,7] and our own experience will result in about one third of the sequences being effective at downregulation to some extent. However, nearly all shRNAs have cytotoxicity in primary peripheral blood lymphocytes that is non-target specific, even when directed to irrelevant sequences such as those of lacZ and luciferase [16]. The cytotoxic effect is dependent on the expression of relatively higher levels of shRNA. Lower expression levels eliminate or reduce cytotoxicity, but also reduce the potency of downregulation. The mechanism of the cytotoxicity was in part due to apoptosis. In other studies, high level expression of shRNAs from adeno associated vectors in mouse livers induced dysfunction in miRNA biogenesis and caused fatality in mice [17]. Thus, the identification of RNAi reagents that are non-cytotoxic yet maintain potency is a critical issue for therapeutic settings where siRNAs are expressed long term.

We previously identified several shRNAs that could effectively downregulate CCR5 [18-20]. However, the expression of these shRNAs from a highly active U6 promoter resulted in cytotoxicity in primary peripheral blood T-cells but not in T-cell lines[19]. Expression from a less active H1 promoter reduced or eliminated cytotoxicity; unfortunately, these shRNAs also were reduced in potency. As such, it was necessary to develop a means to identify shRNAs which are both potent and have no cytotoxicity on primary human T-cells, if these reagents are ever to be utilized in humans.

Here, we demonstrate the selection of an shRNA from a library specific to CCR5 that maintains both potency and lack of cytotoxicity when expressed within primary human PBMC. We characterize this shRNA in regards to its downregulation of CCR5 and target sequence specificity.

## Results

### CCR5 shRNA expressed from U6 promoter are effective but cytotoxic to primary PBMCs

Using published algorithms[7] to predict potent shRNA sequences, we tested 8 shRNAs expressed from the U6 promoter and directed to CCR5 (Table 1). Of these, six showed downregulation of CCR5 in MAGI-CCR5 cells[21], ranging from 2 to greater than 10-fold. The best four of these shRNAs were expressed using a lentiviral vector and demonstrated CCR5 reduction in primary PHA stimulated PBMC. As previously described, cytotoxicity was determined by monitoring the stability of shRNA transduced cells relative to vector transduced cells over a 2 week period of culture [20]. In each case, the fraction of EGFP+ cells (representing transduced cells) declined whereas cells transduced by the vector alone were stable. We previously correlated this cytotoxicity to the greater level of expression from the U6 promoter compared to the H1 promoter[20]. However, while expression from the H1 promoter resulted in little or no loss in the fraction of transduced cells, the extent of CCR5 downregulation was also substantially reduced, rendering these shRNAs inadequate for ablation of CCR5 expression.

**Table 1:** shRNA target sites and the efficiency of CCR5 reduction in MAGI-CCR5 cells

Target region	Target sequence	Nucleotide position	CCR5 reduction on MAGI-CCR5
CCR5-1	aagtgtcaagtccaatctatgac	13	+++

CCR5-2	aagagcatgactgacatctacct	186	++

CCR5-3	ctgacaatcgataggtacctggc	366	-

CCR5-4	gtgacaagtgtgatcacttgggt	442	-

CCR5-5	ttgtcatggtcatctgctactgg	624	++

CCR5-6	cagtagctctaacaggttggaca	809	+

CCR5-7	aaggtcttcattacacctgcagc	517	+/-

CCR5-8	aagttcagaaactacctcttagt	909	+++

Eight shRNAs against human CCR5 were selected by a published algorithm[7]. shRNA target sequences are shown in the second column. Corresponding nucleotide positions of the target sequence in CCR5 mRNA are shown in the 3^rd ^column. MAGI-CCR5 cells were transduced with lentiviral vectors expressing shRNA under the U6 promoter. To determine the expression levels of CCR5 on cell surface, the cells were cultured for 4 days and stained with APC conjugated anti-human CCR5 monoclonal antibody. The reduction levels of in EGFP+ population was analyzed by flow cytometry and shown as following criteria (+++ more than 10 fold reduction, ++ 10 fold reduction, + 3–5 fold reduction, +/- 2 fold reduction, – no reduction).

### Construction of a CCR5 shRNA library and identification of a potent CCR5 shRNA expressed by the H1 promoter

Since screening for several shRNAs predicted by commercially available algorithms were ineffective to downregulate CCR5 without causing cytotoxicity, we adopted a different approach – constructing an shRNA library[22] representing all predicted shRNAs directed against CCR5 and expressed by the H1 promoter. 3000 independent clones were generated. Sequence analysis of 12 were randomly selected clones confirmed the random representation of the library for CCR5 sequences. The shRNA sequences of the library were tested by recloning into a lentiviral vector followed by transduction into CEM cells engineered to ectopically express high levels of CCR5[23]. CCR5 downregulation was monitored by flow cytometry. Out of 380 sequences screened from the library, we identified a single CCR5 shRNA [CCR5 shRNA (1005)] (target sequence- 5'GAGCAAGCUCAGUUUACACC3') which effectively downregulated CCR5 in CEM.NKR-CCR5 cells. This CCR5 shRNA was also effective in downregulating CCR5 in PBMC, with levels of downregulation comparable to or greater than that observed with previous CCR5 shRNA expressed from the U6 promoter.

### Comparison of CCR5 shRNA1005 expression from H1 versus U6 promoter

**Figure 1:**
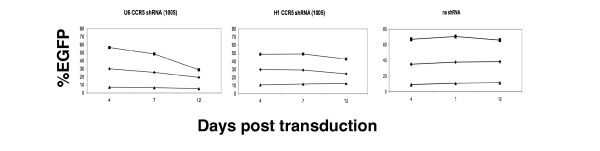
**CCR5 shRNA (1005) is less cytotoxic to PBMC by expressing from the H1 promoter**. PHA/IL-2-stimulated PBMCs (4 × 10^5^) were transduced at m.o.i. of 5, 1 or 0.2 with lentiviral vectors encoding CCR5 shRNA (1005) under the H1 [H1 CCR5shRNA (1005)] or the U6 promoter [U6 CCR5 shRNA (1005)] or no shRNA (no shRNA). The expression levels of EGFP were monitored by flow cytometry at day 4, 7 and 12 post transduction. Rectangle, m.o.i. = 5; diamond, m.o.i. = 1; triangle, m.o.i. = 0.2.

Our previous results indicate that the higher levels of shRNA expression driven by the U6 promoter resulted in cytotoxicity. Since CCR5 shRNA (1005) is expressed by the H1 promoter, we wanted to know whether it also exhibited reduced cytotoxicity. We determined whether CCR5 shRNA (1005) had toxicity to primary T-cells (Figure 1). Over time the EGFP+ cells maintained relatively constant indicating no significant cytotoxicity. The lack of apparent cytotoxicity could be due to either the lower expression levels from the H1 promoter or the specific shRNA sequences expressed. When the same shRNA was expressed using the U6 promoter, we observed a significantly greater relative loss of EGFP+ cells. An NCBI Blast search indicated homology only to human CCR5, so the cytotoxicity is not a result of complete homology to other genes, although we cannot exclude off-target effects due to incomplete complementarity. Therefore, the reduced cytotoxicity does not appear to be an intrinsic feature of the sequence of this shRNA but rather due to the lower expression levels sufficient to achieve efficient downregulation.

Since CCR5 shRNA (1005) was selected by a non-conventional means we further determined whether its functional and structural properties resembled that of typical shRNAs.

### Target site sequence of CCR5 shRNA1005

The use of commercial (Dharmacon Research, Inc[24], Invitrogen[25] and published algorithms[26]) and MIT Whitehead Institute[27] siRNA Selection Program, [28-30] to predict effective siRNA sequences did not result in identification of the same sequence which we identified in CCR5 shRNA (1005). Indeed, the sequence identified here has several features not typically found using published prediction algorithms. For example, using the criteria of Reynolds et.al. [29], the sequence of CCR5 shRNA (1005) is a poor candidate for an effective siRNA. The GC content is within the favored 30–52%, however of eight key criteria, six are deficient: having at least 3 A/U base pairs at positions 15–19, an A at position 3, a U at position 10, an G at position 13, presence of A and lack of G, C at position 19.

One algorithm (Sfold[26]) predicted an siRNA sequence similar to the sequence of CCR5 shRNA (1005), but having an additional C at the 5' end and two nucleotides deleted from the 3' end. Another algorithm (Invitrogen, Stealth[25]) predicts a sequence with the additional C at the 5' end and four additional nucleotides at the 3' end. These sequences would not be predicted to be effective as shRNAs as opposed to siRNAs, since pol III expression generally requires a purine nucleotide at the +1 position for efficient transcription.

The stringency of target sequence selection for RNAi activity of CCR5 shRNA (1005) was further demonstrated by constructing an shRNA with deletion of a single nucleotide from the 3' end of the CCR5 shRNA (1005) sense sequence. This resulted in complete loss of activity (data not shown).

### CCR5 downregulation by shRNA 1005 correlates with decreased levels of mRNA

**Figure 2:**
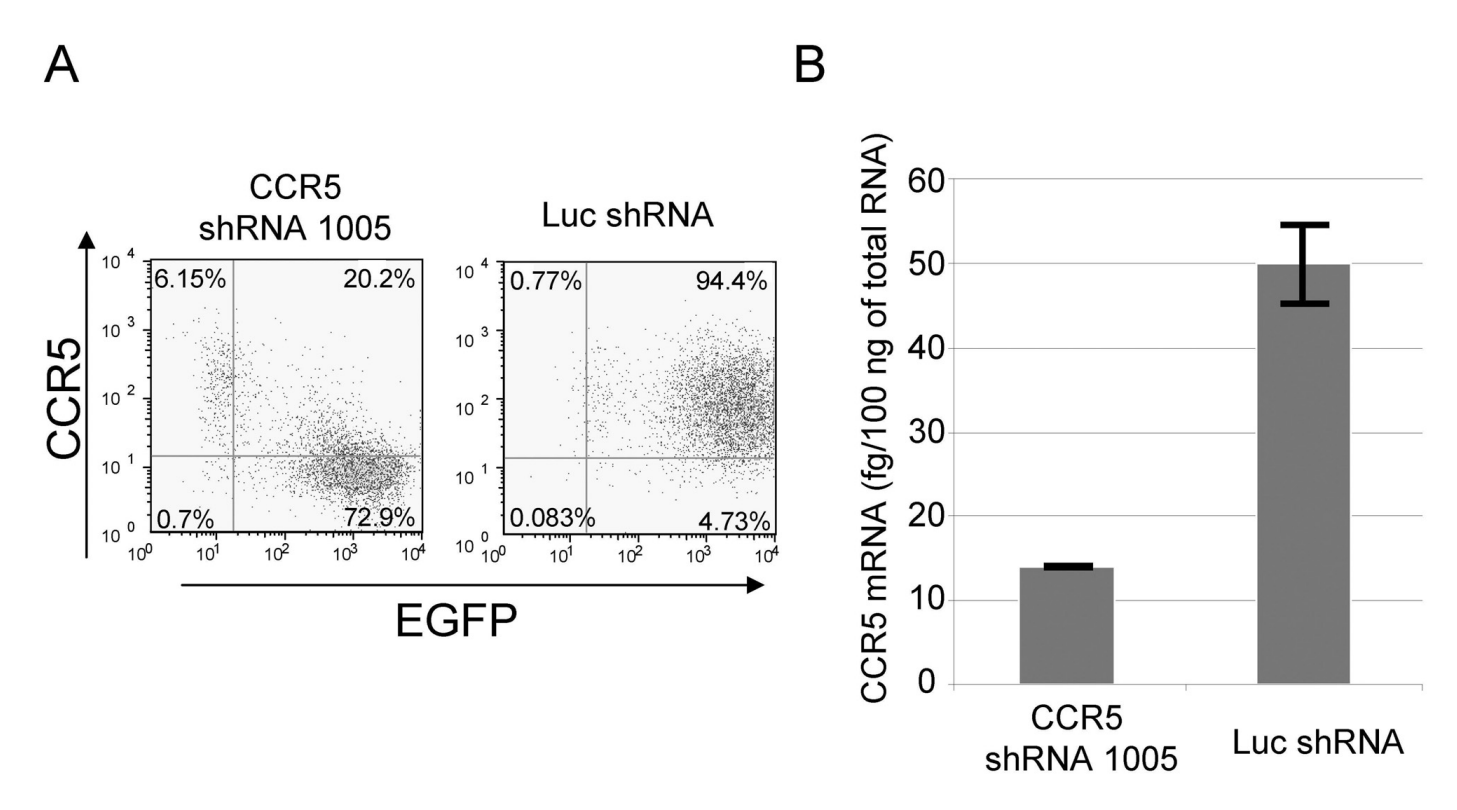
**The decreased levels of CCR5 expression by CCR5 shRNA (1005) correlates with that of CCR5 mRNA**. huCCR5-293T cells were transduced with lentiviral vectors bearing either shRNA 1005 against CCR5 {CCR5shRNA (1005)} or a control shRNA against firefly luciferase (Luc shRNA). To monitor the expression levels of CCR5 on cell surface, the cells were cultured for 4 days and stained with either PE-Cy5 conjugated anti human CCR5 monoclonal antibody or isotype control. CCR5 and EGFP expression were analyzed by flow cytometry. The percentage number in each quadrant is indicated in each panel (A). To measure the levels of CCR5 mRNA, total RNA was isolated using Qiagen RNeasy extraction kit. Quantitative RT-PCR was performed using IQ5 with iScript one step RT-PCR kit using β-actin as an internal control (B).

shRNAs and siRNAs act principally by degradation of the target mRNA [31-33]. We determined whether CCR5 shRNA (1005) acted through a similar mechanism of action by measuring levels of CCR5 mRNA in the presence and absence of CCR5 shRNA (1005) (Figure 2). 293T cells ectopically expressing CCR5 were transduced with CCR5 shRNA (1005) or a control irrelevant shRNA. Downregulation of cell surface CCR5 was observed as expected. Real time RT-PCR was used to measure the levels of mRNA. The mRNA levels decreased approximately 5-fold concordant with the decrease in mean fluorescent intensity of cell surface CCR5 expression. Thus, CCR5 shRNA (1005) acts through a mechanism of action consistent with that of other shRNAs.

### Efficient downregulation of CCR5 shRNA (1005) requires a short hairpin structure

**Figure 3:**
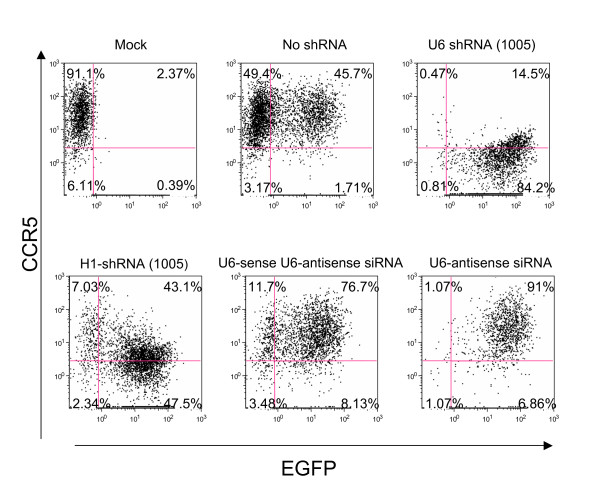
**Efficient reduction requires short hairpin structure and the mode of action is consistent with RNAi**. huCCR5-293T cells (0.5 × 10^5^) were plated into 24 well plates one day before infection. Cells were transduced with lentiviral vectors for 2 hrs in the presence of 8 μg/mL polybrene. The transduced cells were harvested 3 days later and stained with APC conjugated anti-human CCR5 monoclonal antibody for flow cytometry. The efficiency of CCR5 reduction was compared in EGFP+ cells transduced by lentiviral vectors expressing no shRNA (negative control), CCR5 shRNA driven by the U6 {U6-shRNA (1005)} or the H1 promoter {H1-shRNA (1005)}, sense and antisense siRNA expressed from independent U6 promoters from a vector (U6-sense U6-antisense siRNA) or (U6-antisense siRNA). The x axis indicates EGFP expression; the y axis indicates CCR5 expression. The percentage of cells in each quadrant is shown. The quadrant lines were defined by mock-transduction cells. Mock: uninfected cell.

The double stranded stem of the CCR5 shRNA (1005) sequence joined by a 9 nt loop is the structure typically used for shRNA constructs. In some studies, the sense and antisense of the double strand effector siRNA can be expressed independently within the same cell, although the level of activity is generally lower[34]. We tested the independent expression of sense and antisense CCR5 effector sequences in the same vector but with independent promoters (Figure 3). Although we observed very weak downregulation of CCR5 when sense and antisense were both expressed with the U6 promoter, it was considerably less effective than when expressed as a short hairpin. Thus, the short hairpin structure is more effective, presumably because of more efficient formation of double strand siRNA from a hairpin precursor as opposed to kinetic reassociation of sense and antisense following independent expression of each. U6 expression of antisense siRNA sequences alone was completely ineffective, demonstrating that the observed downregulation is not likely to be a result of antisense mechanisms.

### Target sequence specificity

**Figure 4:**
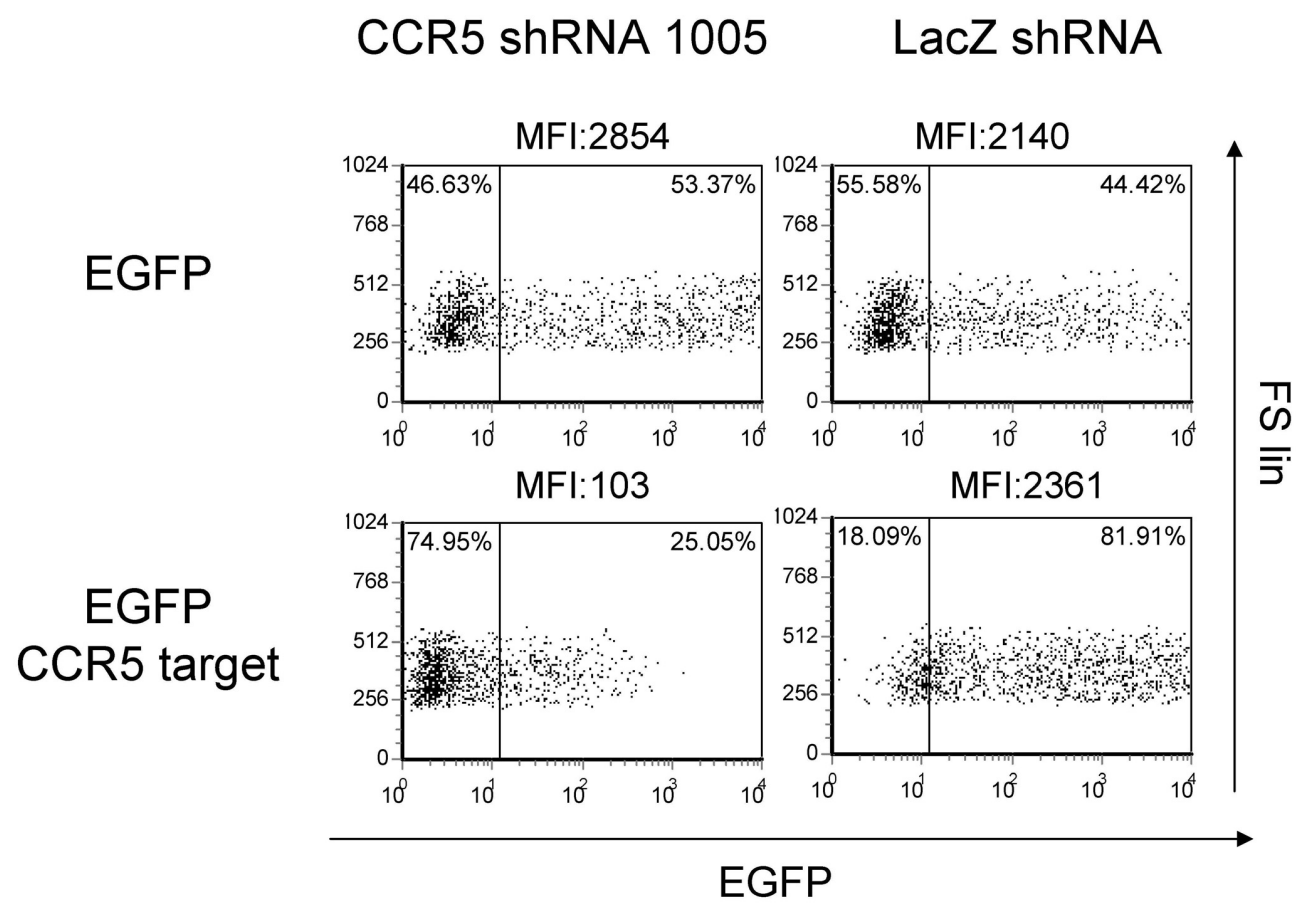
**Target site specific inhibition using a chimeric EGFP-CCR5 siRNA target site (20 nt) fusion mRNA**. 293T cells were co-transfected with CCR5 shRNA (1005) encoding lentiviral vector plasmid DNA and either EGFP-CCR5 siRNA target (EGFP CCR5 target) or EGFP-encoding lentiviral vector (EGFP) by calcium phosphate transfection. The cells were cultured for 2 days and the EGFP expression was measured by flow cytometry. The results are exhibited as forward scatter linear (FS lin) vs EGFP dot plots. The quadrant lines were defined by mock-transfected 293T cells (data not shown) and the percentage numbers are indicated. Mean fluorescent intensity (MFI) of transfected cells is indicated at the top of each panel.

We confirmed that the CCR5 shRNA (1005) acts specifically upon its homologous target sequence by constructing a vector which expresses a chimeric mRNA consisting of an EGFP reporter gene followed immediately by CCR5 sequences of the 20-nucleotide predicted target sequence (Figure 4). Expression of this reporter is specifically ablated in the presence of CCR5 shRNA (1005). Downregulation is dependent upon the presence of the target sequence fused to EGFP; EGFP vectors without the target sequence are not affected in expression of EGFP.

### CCR5 shRNA (1005) sequence mismatch with target sequence ablates activity

**Figure 5:**
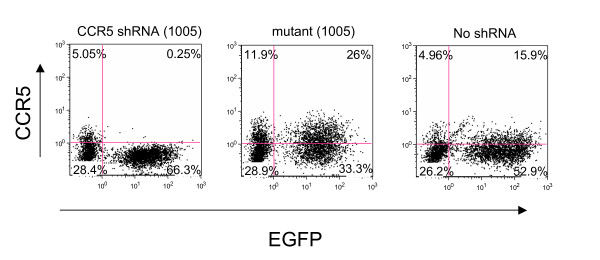
**A 3-nucleotide mutation diminishes the shRNA mediated CCR5 reduction**. PHA/IL-2 activated PBMCs were transduced with a lentiviral vector bearing either shRNA 1005 [CCR5 shRNA (1005)] or the mutant containing 3-nucleotide substitution [mutant (1005)]. To monitor the expression levels of CCR5 on cell surface, the cells were cultured for 12 days and then stained with APC conjugated anti-CCR5 monoclonal antibody. CCR5 and EGFP expression were monitored by flow cytometry. The percentage number in each quadrant is indicated in each panel.

We further assessed the specificity of CCR5 shRNA (1005) by mutating the shRNA within its core domain, disrupting the complete homology with the target sequence (Figure 5). A 3-nucleotide substitution rendered the shRNA incapable of downregulating CCR5 as assayed by cell surface loss of CCR5 expression in primary PHA stimulated PBMC. Thus, complete homology between the siRNA and target sequence is required for activity, consistent with RNAi mechanisms of action.

### HIV-1 inhibition by CCR5 shRNA (1005) in human PBMC

**Figure 6:**
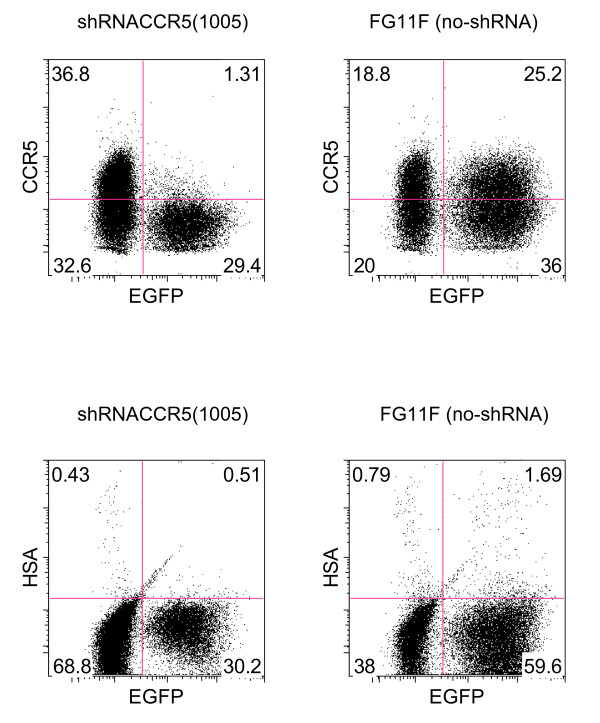
**shRNA CCR5 (1005) inhibit R5 tropic HIV-1 replication in PBMCs**. CD8+ cells depleted PBMCs were activated with PHA for 2 days. Cells were transduced with either shRNA CCR5 (1005) expressing vector or non-shRNA control vector (FG11F). Vector transduced cells were cultured in IL-2 containing medium and infected with either R5 tropic reporter HIV-1_NFNSXHSA _(A). After 3 days infection, cells were harvested and examined for CCR5 expression in EGFP+ vector transduced population in the upper panel, and HSA expression in EGFP+ vector transduced population by flow cytometry.

To examine HIV-1 inhibition by CCR5 shRNA (1005), we transduced PHA/IL2 activated, CD8+ cell- depleted PBMC with the vector expressing CCR5 shRNA (1005) from the H1 promoter. CCR5 shRNA (1005) efficiently reduced CCR5 expression in the EGFP+ population (Figure 6). Transduced cells were infected with the CCR5 tropic reporter HIV-1_NFNSXHSA_. These reporter viruses were modified to express murine heat stable antigen (HSA) cell surface marker gene in place of the HIV-1 accessory gene Vpr, which allows the detection of HIV-1 infected cells (HSA+) by flow cytometry. We stained HIV infected cells with monoclonal antibodies against HSA and examined HSA expression in EGFP+ population. HSA expression was inhibited in the EGFP+ population in the CCR5 shRNA (1005) vector transduced PBMC, indicating CCR5 reduction induced by CCR5 shRNA (1005) was sufficient to inhibit HIV infection. In contrast, HSA expression was not inhibited in the EGFP+ population in the non-shRNA expressing control vector (FG11F) transduced PBMC. These results demonstrated inhibition of CCR5 tropic HIV-1 by CCR5 shRNA (1005).

## Discussion

In this report, we demonstrate that a potent shRNA directed to CCR5 with minimal cytotoxicity can be selected from a library of CCR5 target sequences. This shRNA is relatively rare, identified after screening 380 sequences, as opposed to a frequency of approximately 1 out of 3 effective shRNAs identified through conventional shRNA prediction algorithms. Similar to other shRNAs selected by more conventional means, this shRNA is dependent upon the double strand structure of the RNA and has specific target site specificity. Most importantly, this shRNA shows no obvious cytotoxicity and is effective at downregulating CCR5 in primary cells.

The frequency at which we obtained CCR5 shRNA (1005) was considerably lower than that using conventional algorithms. These algorithms predict potentially potent shRNA expressed using efficient expression vectors or direct transfection of chemically synthesized siRNA; in both cases, high levels of siRNA are present within the cells. Under those conditions, in our hands, all RNAi sequences identified have some cytotoxicity in sensitive primary human lymphocytes when expressed from a U6 promoter. We deliberately screened for potent shRNAs that have low levels of expression, hypothesizing that those RNAs should have lower cellular toxicity. It is noteworthy that the sequence of CCR5 shRNA (1005) does not fit most of the favored rules for identification of effective shRNAs, suggesting that shRNAs obtained by functional screening for potency at lower levels of expression may have different sequence requirements. We have not characterized the specific siRNA species derived within the cells by DICER processing of the shRNA. A related shRNA bearing a single nucleotide substitution and with the same loop sequence was processed correctly to a 22 nucleotide species in rhesus macaque PBMC[19].

Thus, under experimental conditions where cytotoxicity may be an issue, identification of shRNAs that maintain potency, but with reduced cytotoxicity is possible, but considerably more sequences will need to be assayed. Nevertheless, these studies provide confidence that effective shRNAs can be obtained for long term therapeutic purposes such as for use in stem cell gene therapy.

## Conclusion

We characterized the function and structure of a potent shRNA against CCR5 selected by library screening. This shRNA has unique characteristics in regards to its function and structure. The sequence of CCR5 shRNA (1005) did not fit most of the favored rules for identification of effective shRNAs, suggesting that shRNAs obtained by functional screening for potency at lower levels of expression may have different sequence requirements.

## Methods

### Vector construction

The lentiviral vector encoding CCR5 shRNA (1005) under the human H1 RNA polymerase III promoter was previously described[19].

To express CCR5 shRNA (1005) from the human U6 RNA Polymerase III promoter, two complementary DNA oligos, sense 5'-GATCCCCGAGCAAGCTCAGTTTACACCTTGTCCGACGGTGTAAACTGAGCTTGCTCTTTTTC-3', antisense 5'-TCGAGAAAAAGAGCAAGCTCAGTTTACACCGTCGGACAAGGTGTAAACTGAGCTTGCTCGGG-3', were synthesized, annealed, and inserted between *Bbs*I and *Xho*I sites downstream of the U6 promoter of pBS-hU6 plasmid DNA[18] {designed as pBS-hU6 CCR5 shRNA (1005)}. The DNA fragment containing U6 promoter and CCR5 shRNA (1005) was excited from pBS-hU6 CCR5 shRNA (1005) by *Xba*I/*Xho*I digestion and cloned into the same sites of FG12[18].

To express either sense or antisense strand of CCR5 siRNA (1005) by the U6 promoter, U6 promoter containing either strand was amplified by PCR using following primer pairs: for the antisense strand of CCR5 siRNA (1005); 5'-CTCGAGTCTAGAGAATTCCCCCAGTGGAAAGAC-3' and 5'-GAATTCCTCGAGGCTAGCAAAAAGAGCAAGCTCAGTTTACACCGGTGTTTCGTCCTTTCCAC-3', for the sense strand of CCR5 siRNA (1005); 5'-CTCGAGTCTAGAGAATTCCCCCAGTGGAAAGAC-3' and 5'-ACTAGTCTCGAGAAAAAGGTGTAAACTGAGCTTGCTCGGTGTTTCGTCCTTTCCAC-3'. The amplified PCR fragments were digested with *Xba*I and *Xho*I and cloned into the corresponding sites of pBS-SKII vector (Stratagene) and FG12.

The lentiviral vector expressing EGFP-CCR5 target chimeric mRNA (EGFP-CCR5 target/FG11F), which contains the 20 nucleotide predicted shRNA target sequence (5'-GAGCAAGCTCAGTTTACACC-3'), was prepared by PCR amplification using the following primers: 5'-gatcggatccccgggtaccggtcgccaccatggtga-3' and 5'-GCATgaattcgatcgggtgtaaactgagcttgctcgcttacttgtacagctcgtccatgcc-3'. The amplified PCR fragment was digested with *BamH*I and *EcoR*I and cloned into the corresponding sites of the FG12 lentiviral vector. For the generation of 3-nt mismatch mutant of CCR5 shRNA (1005) {designated as a mutant (1005)}, entire human H1 promoter DNA in the pBS hH1-3[18] was amplified using following primers: 5'-CTAGACCATGGAATTCGAACGCTGACG-3' and 5'-GGTGGCTCGAGAAAAAGAGCAAGCTCTCGTTACACCGTCGGACAAGGTGTAACGAGAGCTTGCTCGGGGATCCG-3'. The PCR fragment was cloned into the FG12 between the *EcoR*I and *Xho*I sites.

### Cell culture

MAGI-CCR5 cells (AIDS Research and Reference Reagent Program of the National Institutes of Health)[21] were maintained in DMEM supplemented with 10% FBS, 100 U/ml penicillin, 100 μg/ml streptomycin and 2 mM glutamine.

huCCR5-293T cells were created by infecting 293T cells with a VSV-G pseudotyped human CCR5 expressing pBABE-huCCR5 retroviral vector (AIDS Research and Reference Reagent Program of the National Institutes of Health)[35] followed by puromycin selection (1 μg/ml) and maintained in IMDM, 2%FCS and 8% FBS.

Human primary PBMCs were isolated from leukopacks by Ficoll-Paque PLUS (GE Healthcare) purification and stimulated by 2.5 μg/ml of PHA for 2 days. PBMCs were cultured in RPMI 1640 medium containing 20% FCS and 20 units/ml IL-2 (Roche).

### Lentiviral vector production

All lentiviral vectors were produced by calcium phosphate transfection of 293T cells as previously described[18]. The culture supernatants were harvested on day 2 post-transfection and lentiviral vector particles were concentrated 300-fold by ultracentrifugation.

### Lentivirus vector transduction and HIV-1 Infection

huCCR5-293T cells and MAGI-CCR5 cells were plated into 24 well plates one day before infection. PHA/IL-2 activated PBMCs (4 × 10^5^) were plated into 96 well plates. Cells were infected with lentiviral vectors at different MOI depending on experiments for 2 hr in the presence of 8 μg/mL polybrene (Sigma). The infected cells were collected 3–12 days after infection and analyzed for CCR5 and EGFP expression by flow cytometry or infected with reporter HIV-1 as described previously[16].

### Flow cytometry

Cells were stained with monoclonal antibodies against human CCR5 (2D7; BD Biosciences), mouse IgG2a/κ isotype control conjugated with either PE-Cy5 or APC according to the manufacture's instructions. For measuring HIV-1 reporter virus infection, a PE-labeled anti murine HSA mAb (M1/69, PharMingen) was used. The cells were then fixed with 2% formaldehyde, and EGFP and CCR5 expression were monitored on FC500 (Becton Dickinson). The data was analyzed by CELLQUEST (Becton Dickinson) or FLOWJO (Tree Star) software.

### Cotransfection

Lentiviral vector encoding EGFP-CCR5 target (1 μg) was cotransfected with either 3 μg of CCR5 shRNA (1005)[19] or LacZ shRNA[20] encoding pBluescript onto 293T cells (1 × 10^5^) in a 12-well plate. FuGENE (Roche) was used for cotransfection according to the manufacturer's protocol. Forty-eight hours posttransfection, cells were analyzed for EGFP expression by flow cytometry.

### Quantitative RT-PCR for CCR5 mRNA

Total RNAs from the infected CEM NKR-CCR5 cells were isolated using the Qiagen RNeasy extraction kit following manufacture's instruction. Quantification of mRNA was performed using IQ5 (BioRad) with iScript one-step RT-PCR kit and the following conditions (50°C, 10 min for RT reaction, 95°C, 5 min for RT inactivation and activation of HotStarTaq DNA Polymerase, 40 cycles of 95°C, 15 sec, 52°C, 30 sec for PCR). RNA standards for CCR5 mRNA quantitation was made by serial dilution of *in vitro *transcribed human CCR5 RNA using T7 RNA polymerase (MEGAscript T7, Ambion). Following primers and proves were used for RT-PCR reactions; For Human CCR5; sense primer: gtccccttctgggctcactat, reverse primer: ccctgtcaagagttgacacattgta, probe: FAM-tccaaagtcccactgggcggcag-BHQ1, For β actin; sense primer: cgagcgcggctacagctt, reverse primer: ccttaatgtcacgcacggatt, probe: HEX-accaccacggccgagcgg-BHQ1.

## Competing interests

The authors never received reimbursements, fees, funding, or salary from an organization that may in any way gain or lose financially from the publication of this paper. The authors never have any stocks or shares in an organization that may in any way gain or lose financially from the publication of this paper. The authors have no competing interests to declare in relation to this paper.

## Authors' contributions

SS and DSA designed and performed cytotoxicity experiments, construction of shRNA and siRNAs, infection, flow analysis and wrote the manuscript. PK performed cytotoxicity experiments and constructed shRNA and siRNAs. JB performed HIV infection experiments. QFX constructed shRNAs. MK constructed CCR5 shRNA 1005-mutant, performed flow analysis and qRT-PCR. KC and SK performed flow analysis, SP examined shRNA sequences using commercial and published algorithms. ISYC designed study and drafted the manuscript. Authors read and approved the final manuscript.
